# Polyoxometalate-Stabilized Silver Nanoparticles and Hybrid Electrode Assembly Using Activated Carbon

**DOI:** 10.3390/nano13152241

**Published:** 2023-08-03

**Authors:** Sara Goberna-Ferrón, Laia Cots, Marta Perxés Perich, Jun-Jie Zhu, Pedro Gómez-Romero

**Affiliations:** 1 Instituto Universitario de Tecnología Química (CSIC-UPV), Universitat Politècnica de València, Avda. De los Naranjos s/n, 46022 Valencia, Spain; 2 Catalan Institute of Nanoscience and Nanotechnology (ICN2), CSIC and BIST, Campus UAB, Bellaterra, 08193 Barcelona, Spainpedro.gomez@icn2.cat (P.G.-R.); 3 Materials Chemistry and Catalysis, Debye Institute for Nanomaterials Science, Utrecht University, 3584 CG Utrecht, The Netherlands

**Keywords:** hybrid nanomaterials, triple hybrid materials, polyoxometalate, Ag nanoparticles, activated carbon, electroactive materials, supercapacitor

## Abstract

The intersection between the field of hybrid materials and that of electrochemistry is a quickly expanding area. Hybrid combinations usually consist of two constituents, but new routes toward more complex and versatile electroactive hybrid designs are quickly emerging. The objective of the present work is to explore novel triple hybrid material integrating polyoxometalates (POMs), silver nanoparticles (Ag^0^ NPs), and activated carbon (AC) and to demonstrate its use as a hybrid electrode in a symmetric supercapacitor. The tri-component nanohybrid (AC/POM-Ag^0^ NPs) was fabricated through the combination of AC with pre-synthesized ∼27 nm POM-protected Ag^0^ NPs (POM-Ag^0^ NPs). The POM-Ag^0^ NPs were prepared using a green electrochemical method and characterized via UV-vis and IR spectroscopy, electron microscopy, dynamic light scattering (DLS), X-ray diffraction (XRD), X-ray photoelectron spectroscopy (XPS), and cyclic voltammetry (CV). Afterward, the AC/POM-Ag^0^ NPs ternary nanocomposite material was constructed and characterized. The electrochemical behavior of AC/POM-Ag^0^ NPs’ modified electrodes reveal that the nanomaterial is electroactive and exhibits a moderately higher specific capacitance (81 F/g after 20 cycles) than bare AC electrodes (75 F/g) in a symmetrical supercapacitor configuration in the voltage range 0 to 0.75 V and 20 mV/s, demonstrating the potential use of this type of tri-component nanohybrid for electrochemical applications.

## 1. Introduction

Multifunctional hybrid materials have consolidated as a very versatile and promising class of materials. The hybrid approach allows for a reinforcing combination of properties of dissimilar components in synergic combinations. The compositions are ample and flexible, including magnetic, semiconductor, organic, inorganic, or metallic materials [1,2,3,4]. Their applications are equally varied, ranging from automotive and structural applications to electronics, biomedical, and energy applications. In this context, the intersection between the area of hybrid materials and electrochemistry is a quickly expanding field with key applications, from sensors to energy storage and conversion [5,6]. Regarding energy storage, different devices based on hybrid materials have been recently developed including aqueous Zn-ion batteries [7], lithium–selenium batteries [8], supercapacitors [9,10,11], or ammonium-ion batteries [12]. Various strategies have been made to enhance the electrochemical performance of energy storage systems, from hybrid materials to hybrid devices, providing enhanced energy and power densities by combining battery and supercapacitor materials and storage mechanisms [13]. Hybrid combinations of electroactive and conductive materials for energy storage applications include a wide variety of inorganic species, from extended oxides (or oxyanion phases) to polyoxometalate (POM) clusters, but also conducting organic polymers or carbon materials [14]. Frequently, these hybrid combinations consist of two constituents, but the path to further complexity is also beginning to be explored. For example, triple hybrid materials integrating conducting polymers, oxides, and inorganic molecules [15]; ternary hybrids of MXene, activated carbon (AC), and POMs [16]; ternary nanocomposites of single-walled carbon nanotubes, polyaniline, and silver nanoparticles (NPs) [17]; or electrocatalytic films of conducting polymer/POM/Pt NPs grown layer by layer [18]. Thus, multimaterial combinations with novel nanoarchitectures may provide a differential advantage when it comes to fully exploiting the properties of intrinsically but only partly excellent electrode materials.

POMs are oxoanion clusters of early transition metals (especially V, Mo, and W) that can range from 1 to 5 nm in size and frequently present a rich and reversible redox chemistry. POMs display an extraordinary range of physicochemical properties and are some of the most promising building blocks for functional nanomaterials [19]. Although these clusters can be used for a wide range of applications, the most important functionality of POMs involves their unique electrochemical behavior [20]. Due to the high stability of their redox states, they can participate in fast reversible multielectron transfer reactions, making them potential candidates to achieve a high capacity for energy storage applications [21]. However, they usually exist as salts and solid acids with negligible electronic conductivity and high solubility. Thus, their use as electrode materials requires anchoring onto a variety of conducting substrates such as conducting polymers [22], or carbon materials including nanocarbons or activated carbons (AC) as matrices [23]. AC is a general term commonly applied to a wide family of amorphous carbon materials with excellent and unique textural properties (i.e., specific surface area, porosity, and pore size distribution) [24]. In recent decades, special attention has been paid to the use of ACs as low-cost electrode materials in electrochemical energy storage and conversion devices, mainly supercapacitors [25], lithium and sodium ion batteries [26], and fuel cells [27]. Amongst other aspects, electrical conductivity generally plays a crucial role in the suitability and performance of ACs as electrode materials.

Noble metals are promising for (photo-)electrochemical applications, because of their good conductivity, plasmonic behavior, and chemical stability [28]. However, due to the scarcity and high cost of noble metals, their integration with other sustainable and available materials is considered one of the most attractive ways to optimize their properties and minimize their consumption. Additionally, using metal nanostructures (i.e., NPs) increases the overall surface-to-volume ratio and hence reduces the metal loading. In fact, noble metal NPs have been widely used as conductive dopants in electrode materials for energy storage [29]. Silver has attracted special attention because of its unique physical properties, electrical and thermal conductivity, and low toxicity. Among noble metals, silver is very attractive for electrochemical applications because of its higher conductivity and relatively lower cost [30]. Interestingly, POMs have been exploited for directing the synthesis of metallic NPs (M^0^ NPs) [31,32,33]. POM-protected M^0^ NPs have received enormous attention because of their unique chemical and physical properties with applications in (electro)catalysis [34,35], electrochemical biosensors [36], and biomedicine [37]. However, their use in electrochemical energy storage remains largely unexplored. To the best of our knowledge, there is only one example of an electrode material based on POM-protected Ag^0^ NPs for Li-ion batteries [38].

Herein, POM clusters, Ag^0^ NPs, and AC materials are combined into a single entity through nanoengineering. The strategy to prepare the novel tri-component nanohybrid material is to combine AC and POM-protected Ag^0^ NPs (POM-Ag^0^ NPs), pre-synthesized using a green method in which the POM acts both as a reductant and as a stabilizing agent. First, the synthesis of POM-Ag^0^ NPs was optimized by studying the effects of the reactant’s concentration and the redox potential of the selected POM on the size of the formed NPs. Then, the AC/POM-Ag^0^ NPs tri-component nanohybrid was prepared and fully characterized. Finally, the electrochemical performance of the nanohybrid material was tested, and our results revealed that the nanocomposite is electroactive and exhibits an appreciably improved performance compared with bare AC in a symmetrical supercapacitor configuration due to the synergistic effect of the three components in the final nanocomposite.

## 2. Materials and Methods

### 2.1. Synthesis of POM-Ag^0^ NPs

#### 2.1.1. Preparation of Reduced POM Solutions

The POMs selected for this work are Phosphotungstic acid H_3_[PW_12_O_40_] (abbreviated PW_12_^3−^) and silicotungstic acid H_4_[SiW_12_O_40_] (abbreviated SiW_12_^4−^), both from Sigma-Aldrich (St. Louis, MO, USA) (≥99.9% trace metals basis). First, POM solutions were electrochemically reduced. In a typical experiment, an aqueous solution of POM (50 mL, 1 mM) without any supporting electrolyte was placed in a bulk electrolysis cell (from BASi). A coiled platinum wire (within a fritted glass isolation chamber) and Ag/AgCl electrode were used as counter and reference electrodes. Bulk electrolysis was performed with a BioLogic potentiostat applying the necessary potential to reduce the POM by 1 or 2 e^−^ until intensity values reached a plateau. Potential O_2_ interference was kept to a minimum with vigorous Ar blanketing over the entire process. Upon electrolysis, the solution turned blue due to the formation of reduced POM (_red_POM). The applied potential was selected at the end of the corresponding 1e^−^ or 2e^−^ cathodic peak and before the following reaction’s half-wave potential; that way, the kinetics of the desired reaction are favored while preventing the initiation of the next reduction (Figure S1). The concentration of _red_POM was measured precisely in each case using UV-vis absorption spectrometry (Figure S2) by measuring the characteristic absorbance of the blue _red_POM species (PW_12_O_40_^4−^ ε752nm = 2000 M^−1^ cm^−1^; PW_12_O_40_^5−^ ε653.6nm = 4400 M^−1^ cm^−1^; and SiW_12_O_40_^6−^ ε625 nm = 3800 M^−1^ cm^−1^) [39].

#### 2.1.2. Synthesis of NPs

Silver nanoparticles were obtained by injecting 1mL of a de-aerated aqueous stock solution of AgNO_3_ (Sigma-Aldrich, 99.9999% trace metals basis) into the freshly prepared _red_POM solution (45 mL) that served as both the reductant and protecting ligand. The AgNO_3_ stock solution concentration was varied from 4.05 to 405 mM to obtain final Ag^+^/_red_POM ratios from 0.1 to 10. The initially blue solutions turned green and finally yellow, orange, or brown depending on the Ag^+^/_red_POM ratio utilized (Figure S3), which is indicative of NP formation, within a period between seconds and minutes. The solutions were stirred under Ar atmosphere for 1 h and allowed to stand for 4 days in closed 50 mL centrifuge tubes and then filtered using a 0.45 μm syringe filter before UV-vis, microscopy, and DLS characterization. The residual free POM and unreacted Ag^+^ in solution were removed using centrifugation (at 10,500 rpm, 30 min). The supernatant was removed via pipette and an equal volume of pure water was added in its place. The process was repeated 3 times and the final collected POM–Ag^0^ NPs were re-dispersed into water for characterization (XRD, XPS, IR, and CV) and for the preparation of POM–Ag^0^ NPs/AC hybrids.

### 2.2. Preparation of Hybrid Material

POM-Ag^0^ NPs synthesized using a ratio of Ag^+^/_red_POM = 1 (_red_POM = PW_12_^4−^) were used to prepare POM–Ag^0^ NPs/AC hybrids. The NPs were re-dispersed in water (half of the initial reaction volume) and 60 mg of activated carbon (Cabot Corporation, Alpharetta, GA, USA) was added to yield a concentration of AC of 3mg/mL. The mixture was left under vigorous stirring in a closed flask overnight. Then, the solution was vacuum filtered using a 45 μm filter and washed thoroughly with water. The solid was dried under vacuum at 80 °C.

### 2.3. Preparation of Hybrid Electrodes and Assembly of Coin Cells

The conventional electrodes were fabricated by mixing the active material (AC or AC/POM–Ag^0^ NPs), carbon black, and PVDF (poly(vinylidene fluoride), average molecule weight ≈ 534,000 from Sigma-Aldrich) at a weight ratio of 85:5:10. The mixture was first formed as a slurry by adding a few drops of N-methyl-2-pyrrolidone (anhydrous, ≥99.5%, Sigma-Aldrich), and then coated onto aluminum foil (>99%, 18 µm, Goodfellow, Hamburg, Germany) and dried under vacuum at 120 °C for 12 h. The thickness of the coated POM–Ag^0^ NPs/AC layer on Al foil was 53.25 µm and the loading masses of both electrodes were 4.5 mg. The thickness of the coated AC layer was 20 µm with loading masses of around 3 mg. CR2032 coin cells were used to fabricate symmetric supercapacitors. All the electrodes were pressed at 3MPa before assembling. Additionally, 1 M H_2_SO_4_ served as the electrolyte. Cyclic polarization (CP) was performed in a two-electrode cell to evaluate the capacitive performance in devices. The calculation of capacitance is presented in Supplementary Materials Equation (S1).

### 2.4. Instrumentation

All the electrochemical tests were conducted on Biologic VMP3 multichannel potentiostat. Conventional cyclic voltammetry (CV) was performed in a three-electrode configuration.

UV-vis absorption spectrum was recorded in the range of 350–900 nm using a Cary 4000 spectrophotometer (Agilent Technologies, Santa Clara, CA, USA) and disposable PMMA cuvettes (transmittance range between 300 and 900 nm). Attenuated total reflectance Fourier transform infrared spectroscopy (ATR-FTIR) in the range between 450 and 4000 cm^−1^ using a Bruker Tensor 27 spectrometer (Bruker, Billerica, MA, USA), 32 running scans were collected at a resolution of 4 cm^−1^. The NP dispersion sample was measured by drop-depositing ethanol-suspended NPs on the diamond prism and drying in air before the measurement. UV-vis and ATR-FTIR data were analyzed using Spectragryph optical spectroscopy v1.2.15 software [40].

Dynamic light scattering (DLS) measurements were carried out using Zetasizer Nano ZS and disposable PMMA cuvettes.

Electron microscopy was performed with an FEI Magellan 400L XHR SEM (FEI Company, Hillsboro, OR, USA), operated in bright field STEM mode, with a voltage of 20 kV; water dispersions of NPs were drop-deposited on a 300-mesh copper substrate with a carbon membrane (Lacey Carbon) and evaporated in air for 24 h. Scanning electron microscopy (SEM) images of hybrid materials were obtained using an SEM Quanta 650 FEG (FEI Company, Hillsboro, OR, USA); solid samples were dispersed in ethanol, drop-deposited on a silicon chip, and evaporated in air for 24 h. The silicon chips were previously washed with ethanol, sonicated for a few minutes, and heated at 80 °C for ~1 h. All microscopy images were analyzed using ImageJ v1.53t software [41].

Powder X-ray diffraction (XRD) analyses were performed using an X’Pert PRO PANalytical (Malvern Panalytical, Malvern, UK) with Cu Kα (45 kV and 40 mA) radiation. The samples in solution were prepared with the drop deposition (20 μL) of a filtered ethanol-resuspended solution of nanoparticles on a silicon substrate and air-evaporated for at least 24 h. For hybrid analyses, the solid was mortared for 5 min and directly deposed on the silicon substrate.

X-ray photoelectron spectroscopy (XPS) measurements were performed using a SPECS PHOIBOS 150 hemispherical analyzer (SPECS Surface Nano Analysis GmbH, Berlin, Germany) with Al Kα radiation (1486.74 eV). Solid samples were prepared by placing the powders on a Kapton tape. The NP dispersion sample was drop-deposited on a silicon chip and evaporated in air for 24 h before measurement. Data were analyzed using CasaXPS v2.3.16Dev52 software [42].

## 3. Results and Discussion

### 3.1. Synthesis and Morphological Characterization of POM-Ag^0^ NPs

**Scheme 1:**
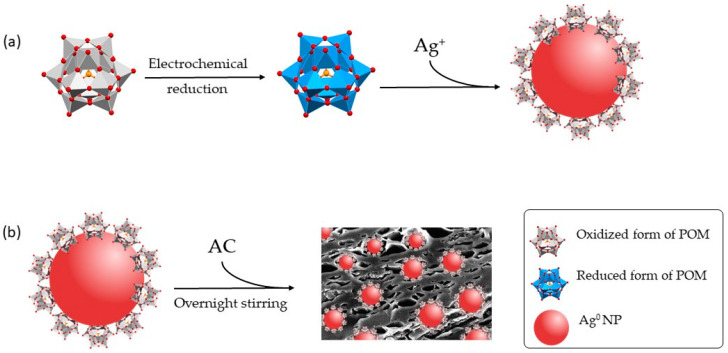
Schematic representation of (**a**) the synthesis of POM-Ag^0^ NPs in which POM serves as both a reducing and stabilizing agent and (**b**) the preparation procedure of the tri-component AC/POM-Ag^0^ NPs hybrid material. Not in scale; POM is 1 nm in size and Ag^0^ NPs are around 27–43 nm depending on the initial reagent concentration.

Reductions of metal salts can be carried out with previously reduced POMs (_red_POM), in which the POM acts as both a reductant and a stabilizing agent [43]. Reduction of POMs can be accomplished photochemically [44], chemically [45], or electrochemically [46]. Among them, the electrochemical method represents a green, facile, and scalable approach. In a simplified view, the silver NP synthesis can be sketched using the following equation (and it is represented in Scheme 1a):

_red_POM + Ag^+^ → _ox_POM + Ag^0^ (1)

Then, in the stabilization process, the adsorption of a layer of POM anions on the nanoparticle surface provides both charge stabilization and steric stabilization. Of course, this reaction is only feasible for metal ions with a higher reduction potential than the reduction potential of the POM used, which is indeed the case for the pair Ag/Ag^+^ (E^0^ = 0.557 V vs. SCE).

#### 3.1.1. Influence of the Concentration of Ag^+^

**Figure 1:**
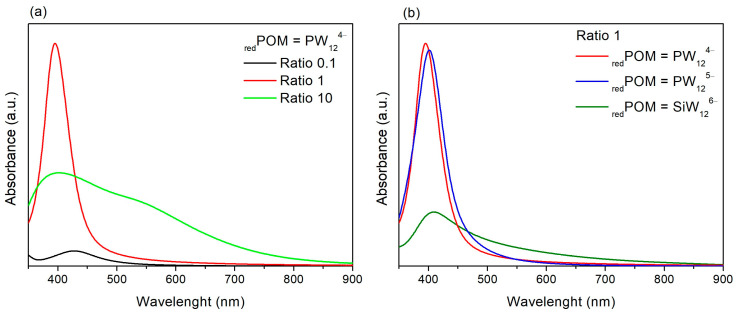
UV-vis spectra of POM-Ag^0^ NPs synthesized by (**a**) changing the initial concentration of Ag^+^ (Different Ag^+/^_red_POM ratios) using PW_12_^4−^ as _red_POM; and (**b**) by changing the reducing power of the used POM while keeping the Ag^+/^_red_POM ratios = 1.

In the first set of experiments, [_red_POM] was kept constant at 1 mM, and the Ag^+/^_red_POM molar ratio values were varied from 0.1 to 10. The POM selected for this work is a heteropoly acid with the chemical formula H_3_[PW_12_O_40_] (with its phosphotungstate anion abbreviated as PW_12_^3−^); the one-electron-reduced anion PW_12_^4−^ was generated electrochemically and used directly as the reducing agent (_red_POM). Figure 1a shows three UV-vis spectra of Ag NPs synthesized by changing the initial concentration of Ag^+^. For Ag^+/^_red_POM = 1, an absorption peak was observed centered at 395.1 nm, featuring a typical surface plasmon resonance associated with spherical or quasi-spherical Ag^0^ NPs [47]. When the ratio is decreased to 0.1, there is a shift to longer wavelengths (427.5 nm) that indicates an increase in the size of the NPs and a decrease in the intensity, indicating a smaller concentration of NPs. For Ag^+/^_red_POM = 10, a broad absorption peak appears at 400.5 nm with an intermediate intensity, which suggests that the NPs have an intermediate size and concentration between ratios 0.1 and 1. Furthermore, a second weak and very broad band indicated via a shoulder at ca. 550 nm emerges, which is characteristic of the coupling of the surface plasmons of aggregated NPs [48].

**Figure 2:**
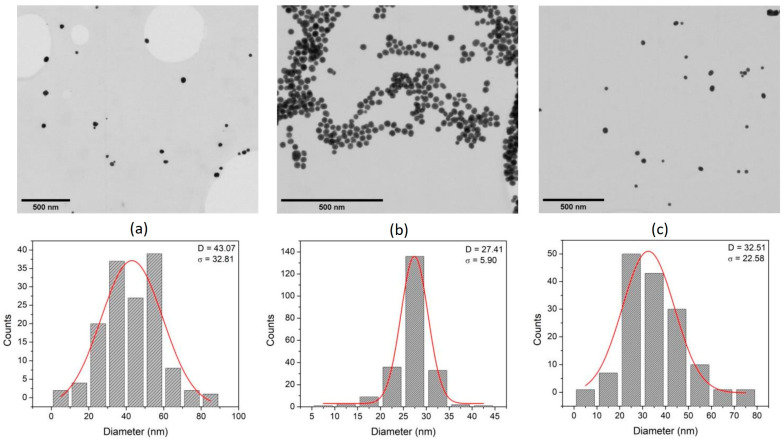
Bright-field STEM images (top) and size distribution (bottom) obtained from the Gaussian fit to the STEM histograms of POM-Ag^0^ NPs synthesized by using Ag^+/^_red_POM ratios of 0.1 (**a**), 1 (**b**), and 10 (**c**). The _red_POM used in the synthesis was PW_12_^4−^.

These results agree with STEM data (Figure 2 and Table 1) where the size distribution obtained from the Gaussian fit to the STEM histograms shows that the size of the NPs follows the trend 0.1 > 10 > 1 of Ag^+/^_red_POM ratios. Aggregation/polydispersity can also be appreciated in the images and reflected in the standard deviation, which is high for ratios 0.1 and 10 compared with ratio 1. Surprisingly, the UV-vis spectra (Figure 1a) for Ag^+/^_red_POM = 0.1 did not show the characteristic band (at 550 nm) of aggregated NPs, probably due to the small concentration indicated by the low intensity of light absorption.

DLS data (Table 1) were also collected for all the NPs. The mean size for Ag^+/^_red_POM ratios 1 and 0.1 agrees with UV-vis and STEM data. However, the DLS for ratio = 10 resulted in a size quality report that did not meet the quality criteria due to a too-polydisperse sample for cumulant analysis, in agreement with the aggregation observed via UV-vis and STEM.

It has been reported that the rate of Ag^+^ reduction strongly affects the initial nucleation of silver particles [49]. A low rate of reduction is achieved for high Ag^+/^_red_POM ratios (low concentration of _red_POM), where crystal growth is favored and leads to larger and more polydisperse or aggregated particles. This is in agreement with our observations for Ag^+/^_red_POM molar ratio = 10.0; where aggregated/polydispersed NPs are observed that are larger than for a ratio = 1. On the other hand, a faster reduction of Ag^+^ (low Ag^+/^_red_POM ratio and high [_red_POM]) should result in smaller and more uniform NPs since the nucleation process is enhanced more than the growth of silver NPs, in agreement with our observations for Ag^+/^_red_POM molar ratio = 1. Following this trend, we would assume that an even faster rate of reaction, as expected for Ag^+/^_red_POM molar ratio = 0.1, would result in even smaller NPs since the nucleation process is faster. However, the low concentration of large polydisperse NPs observed for ratio = 0.1 may be a consequence of two factors that can increase the instability of NPs: (i) Excess equivalents of _red_POM anions can react when exposed to air to give peroxide and eventually hydroxide. These can oxidatively degrade M^0^ NPs [32]. Furthermore, (ii) smaller M^0^ NPs are generally more amenable to oxidations and degradation [49].

Previous studies using other _red_POM and metal salts have shown that varying the initial reagent concentrations may generate different nanostructures (sphere NPs, nanowires, nanoplates, and polygon-shaped nanostructures) [50,51]. However, under the experimental conditions used here, only nanospheres were generated, although with various final concentrations, sizes, and degrees of polydispersity depending on the initial concentration of reactants.

Considering these results, a ratio of Ag^+/^_red_POM = 1 produces spherical NPs with high concentration and low polydispersity. With such a ratio, the influence of the different degree of reduction of the same POM and the influence of the kind of POM from the same series was studied.

#### 3.1.2. Influence of the Reducing Power of the POM

The effect of the degree of reduction of the same POM was studied using the two-electron-reduced PW_12_^5−^, generated electrochemically from PW_12_^3−^, and compared with the one-electron-reduced PW_12_^4−^. The effect of the use of a different POM was performed using another heteropoly acid with the chemical formula H_4_[SiW_12_O_40_] (abbreviated SiW_12_^4−^); the two-electron-reduced SiW_12_^6−^ was generated electrochemically and used directly as _red_POM. In this second set of experiments, [_red_POM] was kept constant at 1 mM, with Ag^+^/_red_POM = 1.

The redox potential (E_0_) values for PW_12_^4−^, PW_12_^5−^, and SiW_12_^6−^ are +0.005, −0.25, and −0.39 V (vs. SCE), respectively [43]. It has been reported that more negative redox potentials lead to faster electron transfer to Ag^+^ and smaller particles [49]. Even though the use of PW_12_^5−^ generated very similar NPs (size, polydispersity, and concentration, see Figure 1b and Figure S4 and Table 1) than the NPs obtained using PW_12_^4−^ as _red_POM agent, the higher negative redox potential of SiW_12_^6−^ generated smaller NPs (DLS size ~12 nm, Table 1) with low concentration and some aggregation indicated by the low-intensity broad UV-vis plasmon peak (Figure 1b); these smaller NPs, however, had a strong tendency to aggregate when they were isolated via centrifugation and re-dispersed in water and STEM measurements could not be obtained. Therefore, the best conditions chosen for the synthesis of Ag NPs using a POM as a reducing and stabilizing agent were: (i) a ratio of Ag^+/^_red_POM = 1 and (ii) the use of PW_12_^4−^ as _red_POM, since a further reduction of the POM to PW_12_^5−^ yielded similar results. POM-Ag^0^ NPs synthesized under these conditions were chosen for further physicochemical characterization.

### 3.2. Physicochemical Characterization of POM-Ag^0^ NPs

#### 3.2.1. Identification of Metallic Silver on POM-Ag^0^ NPs

**Figure 3:**
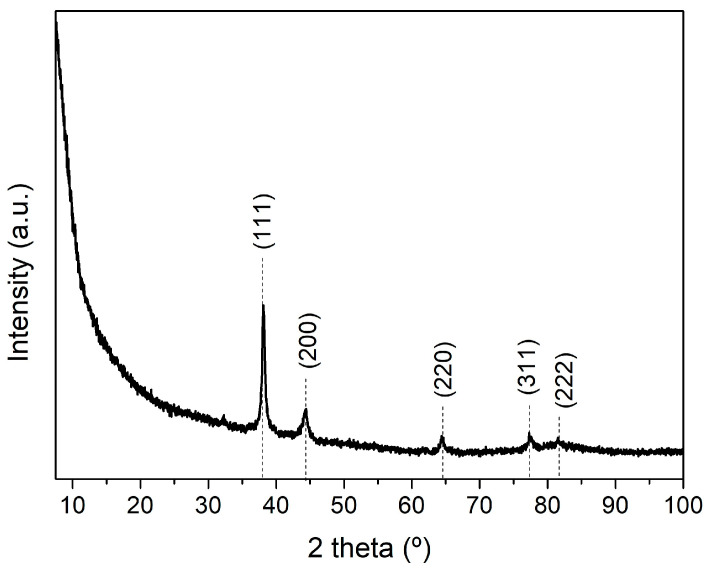
XRD pattern of the synthesized POM-Ag^0^ NPs. Indexed peaks correspond to metallic silver.

XRD analysis was performed to identify the crystallinity of the nanostructures. The XRD pattern recorded from a drop-coated film of the POM-Ag^0^ NPs sample on a silicon substrate is shown in Figure 3. The (111), (200), (220), and (311) Bragg reflections of face-centered cubic (fcc) metallic silver are clearly observed (JCPDS file No. 00-004-0783). The silver peaks are indexed and indicate that the sample studied was highly pure Ag^0^ NPs without any impurities. In addition, an average crystallite size distribution of 24.7 nm was obtained from the XRD data using the Scherrer equation [52], in perfect agreement with the direct measurements of particle size from the STEM images. Finally, particle size measurements using three different techniques (STEM, DLS, and XRD) led to very close values. On the other hand, diffraction peaks corresponding to the PW_12_ structure were not observed; this is common for other POM-M^0^ NPs [50,53,54] and it is most likely related to the lack of an extended long-range order crystal structure of POMs, together with the strong diffraction of the metallic bulk NP compared with the weak dispersion of X-rays with the thin POM layer on the surface.

**Figure 4:**
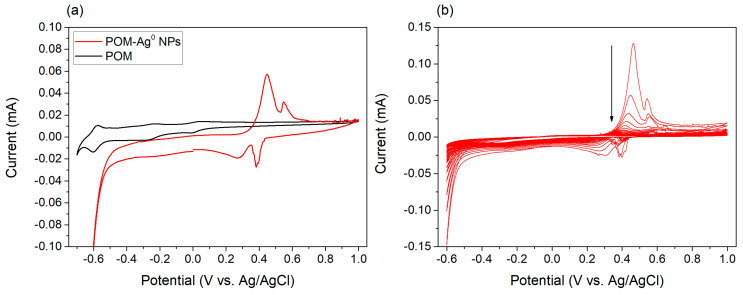
(**a**) Comparative cyclic voltammogram (CV) with glassy carbon electrode modified with free POM (PW_12_^3−^) or POM-Ag^0^ NPs. (**b**) Consecutive CVs of POM-Ag^0^ NPs, the arrow indicates the increase in the CV cycle number. A scan rate of 20 mV/s and 0.5 M H_2_SO_4_ medium were used. Pt wire and Ag/AgCl electrodes were employed as counter and reference electrodes, respectively.

The electrochemical behavior of POM-Ag^0^ NPs was studied using cyclic voltammograms (CVs) from −0.6 to 1.0 V in 0.5 mM sulfuric acid at 20 mV/s on GC electrode drop-casted with the NPs and coated with Nafion. Figure 4a shows CVs corresponding to free POM and POM-Ag^0^ NPs. The CV of the free POM shows the three characteristic redox waves [55]. The CV of POM-Ag^0^ NPs shows an anodic peak at positive potentials that can be attributed to the reversible formation of silver oxides on silver nanoparticles [56,57]. Successive cycling leads to a decrease in the peak currents, (Figure 4b); a possible interpretation could be that the formation of silver oxides on the Ag surface gradually becomes irreversible due to the complete passivation of the metallic surface by a thin layer of insulating oxide. Moreover, the CVs show two weak shoulders, a cathodic one at ca. −0.25 V and an anodic one (even less defined) at ca. 0 V. This may involve a larger polarization than in the free POM, associated with its adsorption in the Ag surface. Furthermore, the peak at −0.25 V vs. Ag/AgCl agrees with the peak observed by Kulesza et al. at 0.0 V vs. RHE [57]. Note that after successive cycling (Figure 4b), the weak shoulder at −0.25 V becomes even more defined, suggesting that POMs remain on the surface of Ag NPs even after complete passivation of the Ag surface. Nevertheless, the weakly defined features of the POM could be a consequence of (i) the diminished potential window due to hydrogen evolution catalyzed with silver [58], and (ii) the thin layer of POMs protecting the surface of Ag^0^ NPs that may not suffice for detection with CV. Therefore, other characterization techniques are used to prove the presence of POMs stabilizing the Ag^0^ NPs.

#### 3.2.2. Presence of POM on POM-Ag^0^ NPs

**Figure 5:**
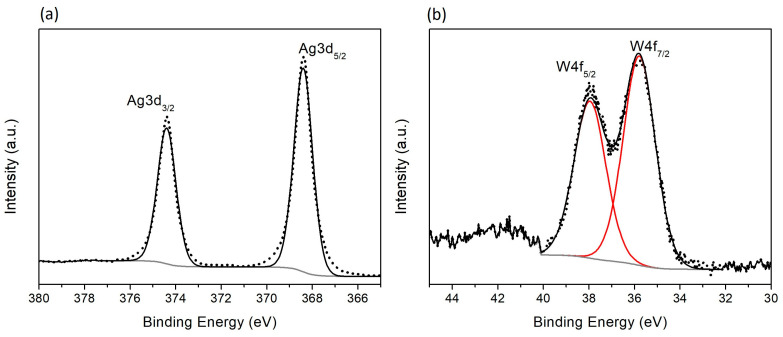
XPS W 4f (**a**) and Ag 3d (**b**) core-level spectra of POM-Ag^0^ NPs. Experimental data (black dots) are shown with envelopes (black lines), component fitting (red lines), and background (grey line).

Figure 5 shows the XPS W 4f and Ag 3d core-level spectra recorded from drop-coated films of the POM-Ag^0^ NPs solution on a silicon chip. The Ag 3d core-level spectrum (Figure 5a) resolves into two spin-orbit components. The Ag 3d_5/2_ and 3d_3/2_ peaks occur at a BE of 368.4 and 374.4 eV, respectively. These values indicate that silver is present only in the metallic form, indicating the formation of Ag^0^ NPs [59]. Figure 5b shows the W4f spectrum recorded from the POM-Ag^0^ film. This spectrum can be resolved with two spin-orbit components with a 4f_7/2_ binding energy (BE) of 35.8 eV which corresponds to W(VI) species [60], pointing to the presence of the fully oxidized PW_12_^3−^ stabilizing the Ag^0^ NPs. The relative atomic composition of the analyzed deposit is 78.2 at % and 21.8 at % of silver and tungsten, respectively (note that XPS is a surface analysis technique and this composition does not correspond to the whole POM-covered Ag^0^ NP but to just a certain depth from the surface). These observations support our proposal that PW_12_ served as both a reducing and an encapsulating molecule.

**Figure 6:**
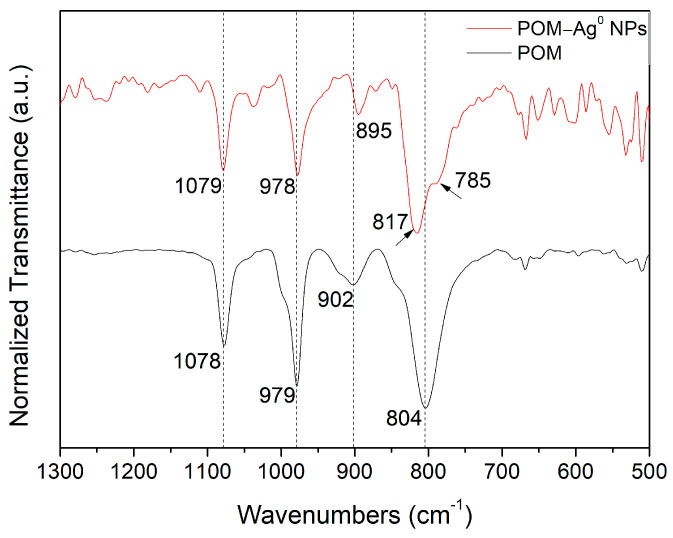
Comparative FTIR spectra of free POM (PW_12_^3−^) and POM-Ag^0^ NPs.

The interaction of POM species with the Ag^0^ NP was studied with FTIR spectroscopy. Figure 6 allows us to compare the spectra of the neat POM (PW_12_) species and their stabilized POM-Ag^0^ NPs. The band positions and assignments based on the literature [61] are indicated in Figure 6. The PW_12_ structure (Keggin structure) contains four types of oxygen atoms: the central oxygen atoms (O_a_), corner-sharing bridging oxygen atoms (O_b_), edge-sharing bridging oxygen atoms (O_c_), and terminal oxygen atoms (O_d_). The central oxygen connects the P to a W atom. The next two types of oxygen atoms bridge two W atoms in adjoining octahedra. Finally, the terminal oxygen atom is bonded to only one W atom [62]. In the spectrum of PW_12_, four bands are observed at 1078, 979, 902, and 804 cm^−1^. The band positions are well correlated with the data in the literature [61]. The spectrum of POM-Ag^0^ NPs shows the presence of the main IR peaks for the fingerprint region of PW_12_, demonstrating the presence of the POM on the silver NP. Furthermore, some differences in the spectra appear due to the presence of the NPs, which suggests that the detected POM was most likely interacting with silver. The band at 804 cm^−1^ splits into two components at 785 and 817 cm^−1^ in the spectrum of POM-Ag^0^ NPs. This band corresponds to a triply degenerated mode involving the W-O_c_-W motion [63]. This band splitting can be rationalized by a decrease in the molecular symmetry of PW_12_ due to the interaction of O_c_ atoms with the silver surface [64]. The band at 902 cm^−1^ involving motions of O_b_ atoms is slightly red-shifted to 895 cm^−1^ in the spectrum of POM-Ag^0^ NPs, which corroborates the lowering of the symmetry due to the interaction of O_b_ atoms with silver. The bands at 1078 and 979 cm^−1^ due to the central O_a_ and terminal O_d_ oxygen atoms, respectively, show negligible shifts, suggesting that these oxygen atoms are not involved in the interaction with silver. Thus, the FTIR spectra indicate that the bridge atoms O_b_ and O_c_ take a part in the interaction of PW_12_ with the Ag^0^ NPs, while the distal O_d_ atoms are farther away from the surface. The participation of the bridge oxygen atoms in the interaction with the silver surface was also observed for Ag^0^ NPs stabilized via Keggin-type silicotungstate anions H_4_[SiW_12_O_40_] (SiW_12_) [64]. Therefore, by using complementary characterization techniques, we have demonstrated the synthesis of pure POM-Ag^0^ NPs where the POM acts both as a reducing and an encapsulating agent.

### 3.3. Preparation and Characterization of AC/POM-Ag^0^ NPs Hybrid Material

#### 3.3.1. Physisorption of POM-Ag^0^ NPs on AC

**Figure 7:**
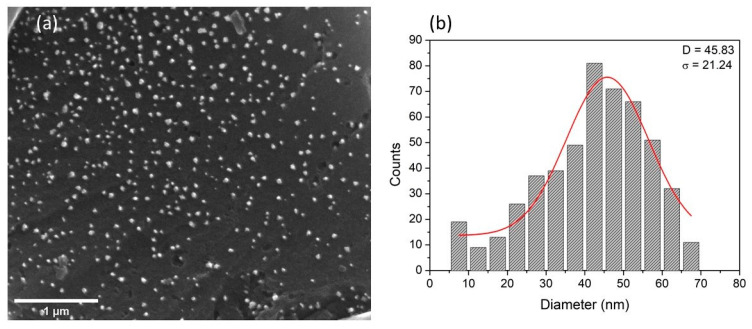
SEM image with a one-micron scale bar showing an even distribution of nanoparticles on AC (**a**) and size distribution (**b**) obtained from the Gaussian fit to the SEM histograms of AC/POM-Ag^0^ NPs nanohybrid material.

POM-Ag^0^ NPs were incorporated into AC to create a tri-component nanohybrid material. The incorporation into AC was performed using a physisorption method (represented in Scheme 1b), depositing pre-made NPs on AC through the vigorous stirring of a suspension. The samples were centrifuged and washed with pure water before subsequent characterization. Figure 7 shows a representative SEM image, where the POM-Ag^0^ NPs are evenly distributed on the AC substrate. However, SEM size distribution data indicates that they tend to aggregate to an average size of 45.83 nm in comparison with the free POM-Ag^0^ NPs (average size of 27.41 mm, Figure 2 and Table 1). This aggregating phenomenon on AC may be attributed to the lack of strong interaction with the support.

**Figure 8:**
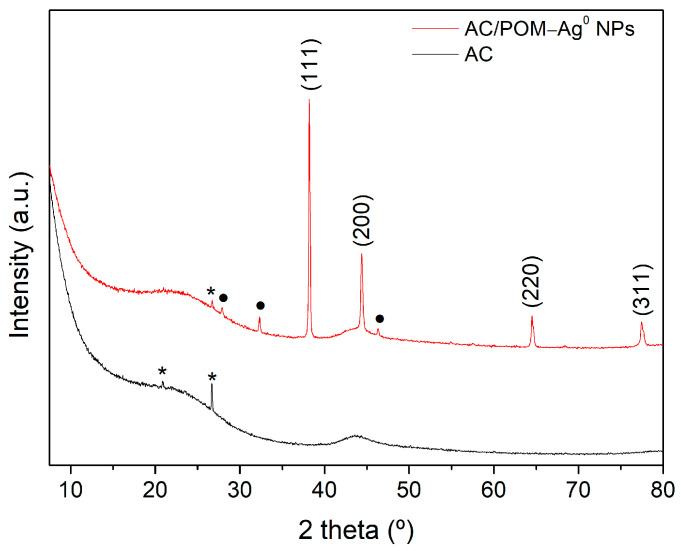
XRD patterns of bare AC (bottom) and AC/POM-Ag^0^ NPs. Small peaks marked with filled circles and asterisks correspond to minor AgCl and SiO_2_ impurities, respectively.

The nanohybrid was further characterized by XRD and XPS. The XRD patterns of pure AC and AC/POM-Ag^0^ NPs are displayed in Figure 8. The AC pattern shows broad peaks of amorphous carbon (2θ = ∼24° and 44°) [65]. After the incorporation of the NPs, the characteristic peaks assigned to the Bragg reflections of metallic Ag fcc structure emerge, suggesting that POM-Ag^0^ NPs have been incorporated into the AC support. As in the case of free POM-Ag^0^ NPs (Figure 3), diffraction peaks corresponding to the PW_12_ structure were not observed. Therefore, the presence of the POM-protecting ligands in the AC/POM-Ag^0^ NPs hybrid material was confirmed using XPS.

**Figure 9:**
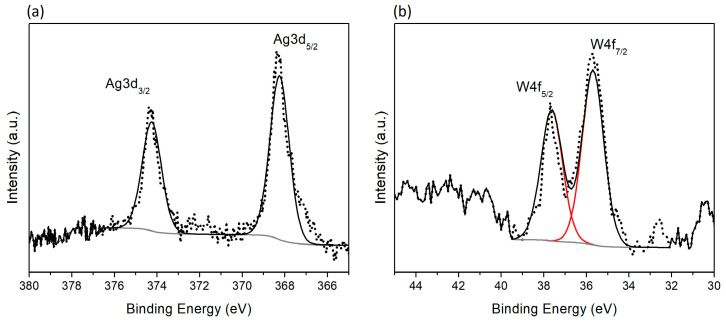
XPS W 4f (**a**) and Ag 3d (**b**) core-level spectra of AC/POM-Ag^0^ NPs. Experimental data (black dots) are shown with envelopes (black lines), component fitting (red lines), and background (grey line).

The Ag 3d_3/2_ and 3d_5/2_ doublet can be easily observed in the XPS spectrum of the as-prepared AC/POM-Ag^0^ NPs hybrids (Figure 9). The 3d_5/2_ level is located at 368.25 eV and that of 3d_3/2_ at 374.25 eV, confirming that silver is present only in the metallic form. The presence of tungsten was also detected, and the W4f_7/2_ and W4f_5/2_ doublet with binding energies of 35.69 and 37.63 eV, respectively. These values indicate that the tungsten is in its fully oxidized form (W^VI^) in the POM, corroborating the formation of ternary hybrids of AC/POM-Ag^0^ NPs. Therefore, the above characterization techniques confirm that POM-Ag^0^ NPs were successfully incorporated homogeneously onto AC to form a tri-component nanohybrid material.

#### 3.3.2. Electrochemical Properties of Hybrid Electrodes

**Figure 10:**
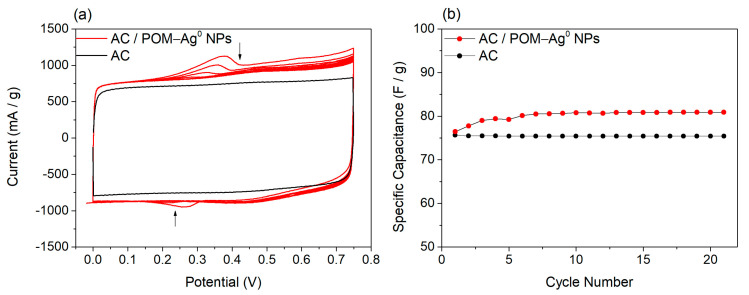
(**a**) CV curves of electrodes in symmetric coin cells using 1M H_2_SO_4_ electrolyte and collected at 20 mV/s, the arrows indicate the increase in the CV cycle number. (**b**) Variation in the specific capacitance as a function of CV cycle number.

The promising application of the AC/POM-Ag^0^ NPs composite as a hybrid electrode was preliminarily tested by evaluating the electrochemical performance. To this aim, symmetric cells were assembled by sandwiching a separator soaked in 1M H_2_SO_4_ electrolyte between two identical electrodes (AC or AC/POM-Ag^0^ NPs). Figure 10a shows cyclic polarization (CP) curves for AC and AC/POM-Ag^0^ NPs symmetric cells at a constant scan rate of 20 mV/s. The shape of the CP curves for the AC symmetric cell is rectangular due to the contribution of the electric double-layer capacitance (EDLC). On the other hand, quasi-rectangular CP curves are observed for the AC/POM-Ag^0^ NPs symmetric cell, with various redox waves originating from Ag^0^ NPs that overlap with a rectangular (capacitive) envelope. This CP structure indicates the coexistence of both charge-storing mechanisms, faradaic and EDLC. It is worth noting that the observed waves from silver become smaller with increasing cycle numbers and become imperceptible, indicating some degree of instability, as observed for free POM-Ag^0^ NPs (see Figure 4b). As discussed above (see Section 3.2.1 and Figure 4b), this may be a consequence of silver oxide formation on the Ag surface with the POMs remaining on the surface of the NPs. In fact, there is a correlation between the decrease in waves from silver (Figure 10a) during the first few cycles with an increase in the specific capacitance calculated from the area in the CP (Figure 10b). This may be explained by the difference in specific capacitance of the thin layer of silver oxide compared to silver [1]. Nevertheless, the specific capacitance is moderately higher for AC/POM-Ag^0^ NPs hybrid electrodes than for bare AC electrodes; while the specific capacitance of AC is 75 F/g, AC/POM-Ag^0^ NPs reach 81 F/g after 20 cycles. This result indicates that the hybrid material is electroactive and exceeds to some degree AC in a symmetrical supercapacitor configuration. The change in the specific capacitance with variations in the scan rate is shown in Figure S5; the specific capacitances of the AC and AC/POM-Ag^0^ NPs cells decrease with increasing current density. For AC/POM-Ag^0^ NPs, the highest value was 83.5 F/g at a scan rate of 2 mV/s, which further decreased to 77.5 F/g at a scan rate of 50 mV/s. This reduction of the specific capacitance at high scan rates has been attributed to the low diffusion of the electrolyte ion. Due to the time constraint, the ionic motion in the electrolyte during the high-rate charge–discharge process is always limited by diffusion, and only the outer active surface is utilized for charge storage. Slowing down the scan rate can allow the electrolyte to penetrate into pores more thoroughly and to make greater contact with the internal surface of the electrode material, resulting in more charge stored on the surface of the electrode (larger capacitance) [66].

Conclusively, this study provides promising preliminary data supporting the use of this type of ternary hybrid materials as electrodes and demonstrating the potential of other POM/M^0^ NPs/nanocarbon composites for electrochemical applications. Furthermore, the possibility of tunning the degree of silver oxidation (i.e., electrochemical pre-treatment) while keeping intact the POM-protecting ligands, opens new interesting routes for other applications such as electrocatalysis.

## 4. Conclusions

In summary, uniform and sized-controlled silver nanoparticles were prepared using a green method in which the POM acts both as a reductant and as a stabilizing agent. Control of the size and homogeneity of the particles was achieved by varying the silver concentration and the redox potential of the POM. Novel ternary AC/POM-Ag^0^ NPs nanohybrids were successfully fabricated using a facile method. Extensive characterization of the new nanomaterial was performed using a range of spectroscopic and imaging techniques, which demonstrated unambiguously that POMs are attached to the Ag^0^ NP surface. The use of this nanocomposite as a hybrid electrode has been explored, and its electrochemical performance reveals that the material is electroactive and exhibits moderately higher performance than bare AC electrodes in a symmetrical supercapacitor configuration. Our results are proof of concept that the electrochemical properties of the tri-component nanohybrid material hold great prospects in electrochemical applications.

Future studies will explore new approaches to improve the stability and cyclability of the hybrid electrodes by developing new covalent and non-covalent linkage modes between POMs and metallic surfaces/nanocarbon. Furthermore, nanoparticle size, shape, and composition can be used to both tailor and improve the properties of new composite materials. In this context, a more systematic study of their synergistic properties and structure–activity relationships will significantly help develop the full potential of hybrid materials, electrodes, and devices for electrochemical applications.

## Figures and Tables

**Table 1:** Summary of the main parameters obtained from UV-vis, STEM, and DLS data.

		UV-vis	STEM Gaussian Fit				DLS	
_red_POM	Ratio Ag^+/^_red_POM	Plasmon Band (nm)	Mean Size (nm)	Std. Dev.	Particle Count	PDI	Mean Size Number (nm)	Mean PDI
PW_12_^4−^	0.1	427.50	43.07	32.81	140	0.58	39.17	0.21
PW_12_^4−^	1	395.10	27.41	5.90	221	0.05	20.40	0.29
PW_12_^4−^	10	400.50	32.51	22.59	143	0.48		
PW_12_^5−^	1	401.10	27.62	11.23	104	0.17	28.62	0.22
SiW_12_^6−^	1	408.00					12.12	0.29

## Data Availability

Data available on request. The data presented in this study are available on request from the corresponding author.

## Supplementary Materials

The following supporting information can be downloaded at: https://www.mdpi.com/article/10.3390/nano13152241/s1, Figure S1: Cyclic Voltammetry results of PW_12_ in water; Figure S2: UV-vis spectroscopy signal of reduced and oxidized states of the POM; Figure S3: Photography of the reaction mixtures resultant from the synthesis of POM-Ag^0^ NPs; Figure S4: STEM image and size distribution of POM-Ag^0^ NPs synthesized using PW_12_^5−^; Figure S5: Variation of specific capacitance of bare AC and AC/POM-Ag^0^ NPs symmetric cells with scan rate; Equation (S1): Calculation of the capacitance. References [55,67] are cited in the Supplementary Materials.
