# Supplementary material for: Polyoxometalate-Stabilized Silver Nanoparticles and Hybrid Electrode Assembly Using Activated Carbon
Source: Nanomaterials (Basel). 2023 Aug 3;13(15):2241. doi: 10.3390/nano13152241 (PMC10421052; doi:10.3390/nano13152241)
Supplement: Supplementary file 1 [file nanomaterials-13-02241-s001.zip › nanomaterials-2524417-supplementary.pdf]

Polyoxometalate-Stabilized Silver Nanoparticles and Hybrid Electrode Assembly using Activated Carbon

Sara Goberna-Ferrón [1,*], Laia Cots [2], Marta Perxés Perich [3], Jun-Jie Zhu [2] and Pedro Gómez-Romero [2]

1 Instituto Universitario de Tecnología Química (CSIC-UPV), Universitat Politècnica de València, Avda. De los Naranjos s/n, 46022 Valencia, Spain
2 Catalan Institute of Nanoscience and Nanotechnology (ICN2), CSIC and BIST, Campus UAB, Bellaterra, 08193 Barcelona, Spain;
3 Materials Chemistry and Catalysis, Debye Institute for Nanomaterials Science, Utrecht University, 3584 CG Utrecht, The Netherlands
* Correspondence: sgobfer@itq.upv.es

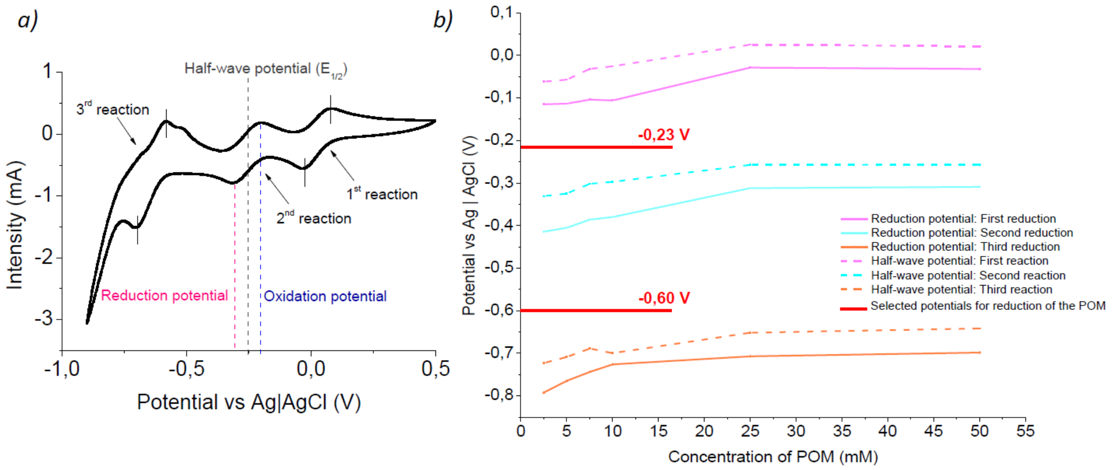

Figure S1. *Cyclic Voltammetry results of POM in water. a) Example of a Cyclic Voltammogram of a 50 mM POM solution at 100 mV/s of scan rate. Three redox waves corresponding to the 1, 2 and 3 e^- reduction reactions are observed in agreement with the literature.[55] b) Change of reduction and half-wave potentials with nominal concentration of POM; values were taken from CV measurements at 100 mV/s of scan rate. Selected values for the chronoamperometric study of the POM are marked in red.*

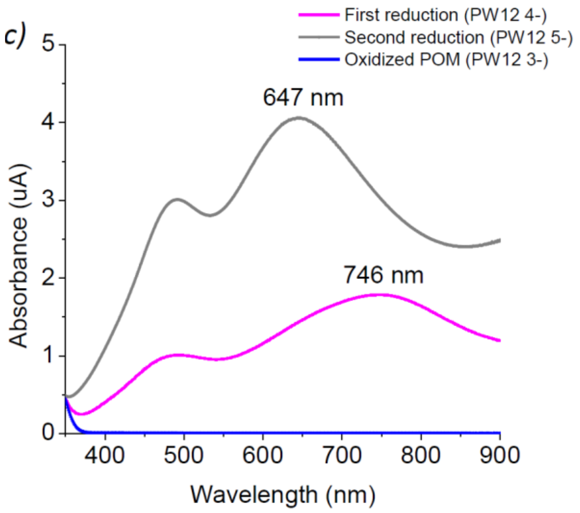

Figure S2. *UV-vis spectroscopy signal of both reduced states (PW_{12}^{4-} and PW_{12}^{5-}) and oxidized state of the POM (PW_{12}^{3-}).*

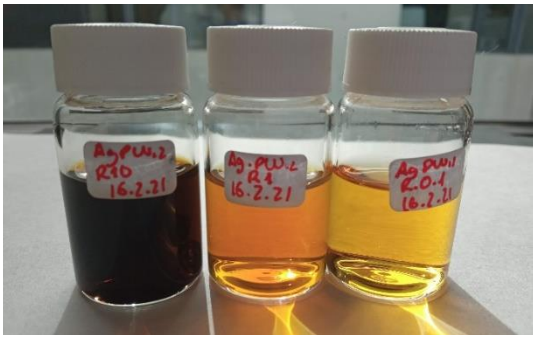

Figure S3. Photography of the reaction mixtures resulting from the synthesis of POM-Ag^0 NPs using Ag^+ / $_{red}$POM ratios of 10 (left), 1 (center), and 0.1 (right).

The capacitance C (F) was calculated from the cyclic polarization curves according to the following equation:[67]

$$C_{cell} = \frac{\int I(V)dV}{2v\Delta V} \qquad \textbf{(Equation S1)}$$

Where C_{cell} is the capacitance of the device, I is the current, ΔV is voltage window, and v is the scan rate.

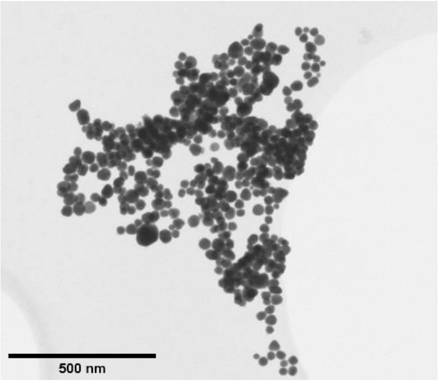

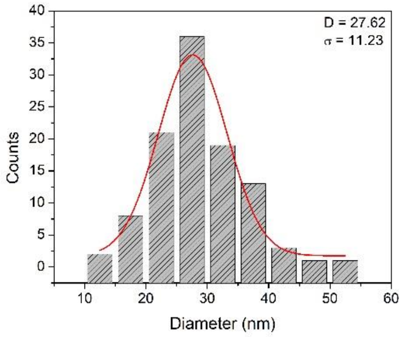

Figure S4. *STEM image (left) and size distribution (right) obtained from Gaussian fit to the STEM histograms of POM-Ag^0 NPs synthetized using: Ag^+ / $_{red}$POM = 1 and $_{red}$POM = PW_{12}^{5-}.*

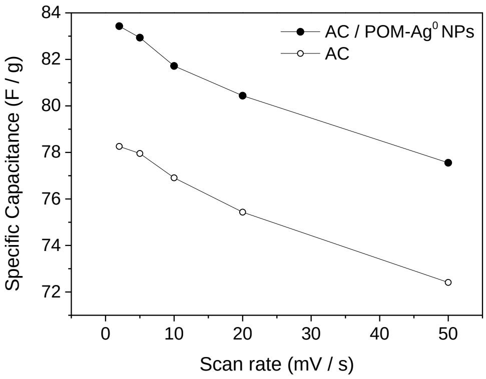

Figure S5. *Variation of specific capacitance of bare AC and AC / POM-Ag^0 NPs symmetric cells with scan rate.*

References:

55. Sadakane, M.; Steckhan, E. Electrochemical Properties of Polyoxometalates as Electrocatalysts. *Chemical Reviews* 1998, *98*, 219–238, doi:10.1021/cr960403a.

67. Allagui, A.; Freeborn, T.J.; Elwakil, A.S.; Maundy, B.J. Reevaluation of Performance of Electric Double-Layer Capacitors from Constant-Current Charge/Discharge and Cyclic Voltammetry. *Sci Rep-uk* 2016, *6*, 38568, doi:10.1038/srep38568.
